# Three‐dimensional genome structure shapes the recombination landscape of chromatin features during female germline stem cell development

**DOI:** 10.1002/ctm2.927

**Published:** 2022-06-22

**Authors:** Geng G. Tian, Changliang Hou, Jing Li, Ji Wu

**Affiliations:** ^1^ Renji Hospital Key Laboratory for the Genetics of Developmental & Neuropsychiatric Disorders (Ministry of Education) Bio‐X Institutes School of Medicine Shanghai Jiao Tong University Shanghai China; ^2^ Department of Bioinformatics and Biostatistics School of Life Sciences and Biotechnology Shanghai Jiao Tong University Shanghai China; ^3^ Key Laboratory of Fertility Preservation and Maintenance of Ministry of Education Ningxia Medical University Yinchuan China

**Keywords:** chromatin structure, FGSC development, Hi‐C

## Abstract

**Background:**

During meiosis of mammalian cells, chromatin undergoes drastic reorganization. However, the dynamics of the three‐dimensional (3D) chromatin structure during the development of female germline stem cells (FGSCs) are poorly understood.

**Methods:**

The high‐throughput chromosome conformation capture technique was used to probe the 3D structure of chromatin in mouse germ cells at each stage of FGSC development.

**Results:**

The global 3D genome was dramatically reorganized during FGSC development. In topologically associating domains, the chromatin structure was weakened in germinal vesicle stage oocytes and still present in meiosis I stage oocytes but had vanished in meiosis II oocytes. This switch between topologically associating domains was related to the biological process of FGSC development. Moreover, we constructed a landscape of chromosome X organization, which showed that the X chromosome occupied a smaller proportion of the active (A) compartment than the autosome during FGSC development. By comparing the high‐order chromatin structure between female and male germline development, we found that 3D genome organization was remodelled by two different potential mechanisms during gamete development, in which interchromosomal interactions, compartments, and topologically associating domain were decreased during FGSC development but reorganized and recovered during spermatogenesis. Finally, we identified conserved chromatin structures between FGSC development and early embryonic development.

**Conclusions:**

These results provide a valuable resource to characterize chromatin organization and for further studies of FGSC development.

## Graphical abstract

1. Hi‐C analysis of meiotic chromatin architecture during FGSC development.

2. TADs were attenuated and then disappeared during FGSC development.

3. X chromosome shows unique chromatin architecture during FGSC development.

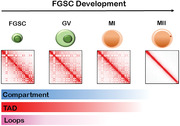

## 1 INTRODUCTION

Proper folding of chromatin is a highly organized process that is crucial for the basic functions of eukaryotic cells.^1^, ^2^ During eukaryotic meiosis, the high‐order chromatin architecture undergoes dramatic remodelling followed by two rounds of segregation^3^, ^4^, ^5^ in which the chromosomes are broken, repaired, and paired with their homologs. Unlike the early discovery of spermatogonial stem cells, the discovery of female germline stem cells (FGSCs) was more recent.^6^, ^7^, ^8^ FGSCs differentiate into mature oocytes in vivo and in vitro.^6^, ^7^, ^8^, ^9^ During this process, FGSCs not only renew themselves but also differentiate into germinal vesicle (GV) oocytes. Then, they initiate entry into the prophase of meiosis I (MI) oocytes with assembly and disassembly of the synaptonemal complex of chromatin and finally into the prophase of meiosis II (MII) oocytes.^8^ This process is called FGSC development. During FGSC development, each stage of transition involves a major reorganization of the chromatin structure, which has led to questions such as what are the characteristics of the chromatin structure, how chromatin organization is remodelled, and what is the relationship between chromatin organization and its development.

Recently, the developed high‐throughput chromosome conformation capture (Hi‐C) technology is so powerful that the genome organization can easily be observed and quantified in the nuclei of cells.^2^ Using this technique, the active/inactive (A/B) compartment status, topologically associating domains (TADs), and chromatin loops were found to be important features of chromatin organization.^2^, ^10^, ^11^ These features are closely related to their biological functions. For example, the A/B compartment status and chromatin loops are associated with gene expression and the distribution of histone modifications, and the disruption of TADs can lead to diseases.^11^, ^12^, ^13^, ^14^ Recently, the landscape of chromatin organization was found to be reorganized during spermatogenesis, whereby TADs and A/B compartments disappear in pachytene spermatocytes (PACs) and recover in sperm.^5^ Polycomb‐associating domains, which are related to the polycomb group proteins that are epigenetic regulators of transcription, have been found in mouse oocytes.^15^ These studies on the roles of the chromatin structure in the development of germline cells have contributed to our understanding of the involved molecular mechanisms. However, the chromatin organization during FGSC development has not been studied until now, and the relationship between FGSC development and early embryonic development remains undiscovered.

In this work, we applied Hi‐C to establish a dynamic landscape of chromatin organization during FGSC development. We found that the global three‐dimensional (3D) genome was dramatically reorganized during FGSC development. Furthermore, TADs were weakened in GV stage oocytes and still present in MI stage oocytes, but they had disappeared in MII oocytes. We compared the chromatin organization between the X chromosome and autosomes and found that the X chromosome had a different compartment status than autosomes, which may be related to X inactivation. Finally, we found that some regions in the allelic genomes of early embryonic development were conserved. These results demonstrated the dynamic change of chromatin organization during FGSC development and described the relationship between FGSC and early embryonic development.

## 2 METHODS

### 2.1 Animals

We obtained C57BL/6J female mice from Shanghai Slac Laboratory Animal Co., Ltd. (Shanghai, China). We performed all experiments followed with the approved protocols, which were passed by the Institutional Animal Care and Use Committee at Shanghai Jiao Tong University.

### 2.2 Collection of mouse oocytes

Six‐week‐old C57BL/6J female mice were intraperitoneally injected with 10‐IU pregnant mare serum gonadotropin (PMSG) and then injected with 5‐IU human chorionic gonadotropin (hCG) 48 h later. Mouse GV oocytes were isolated by puncturing ovaries with a hypodermic needle at 46–48 h after PMSG injection. MI oocytes were isolated from ovaries at 9 h after hCG injection. GV and MI oocytes were distinguished under a microscope as follows: GV oocytes had a visible nucleus, whereas MI oocytes had neither a visible nucleus nor a first polar body. To avoid granulosa contamination, GV and MI oocytes were washed eight times with a minimum essential medium containing 1‐mg/ml hyaluronidase. The zona pellucida was removed using Acidic Tyrode’ s Solution (Sigma), followed by fixation with 1% paraformaldehyde. The GV and MI oocytes were then prepared for Hi‐C experiments.

### 2.3 Hi‐C library generation

The prepared cells were followed with a previously described method of Hi‐C library generation with minor modifications.^16^, ^17^ Briefly, the cells were fixed in 1% formaldehyde and quenched with 2.5‐M glycine. The fixed cells were lysed with lysis buffer and centrifuged to obtain the nuclei. Then, we resuspended the pellets in 0.5% sodium dodecyl sulphate and quenched with 10% Triton X‐100. Next, the nuclei were digested with Mbol restriction enzyme overnight at 37°C and filled with 0.4‐mM biotin‐14‐dATP, 10‐mM dCTP, 10‐mM dGTP, and 10‐mM dTTP. Then, the fragments were ligated for 6 h with rotation at room temperature and the DNA fragments were purified. Subsequently, DNA was sheared to 300–500 bp using a Digital Sonifier (Branson) and pulled down with Dynabeads MyOne Streptavidin T1 beads. Finally, the sequencing library was prepared by NEBNext Q5 Hot Start HiFi polymerase chain reaction (PCR) in accordance with the manufacturer's instructions.

### 2.4 Hi‐C data processing, mapping, iterative correction, and eigenvector decomposition (ICE normalization)

Hi‐C data were processed as described previously.^18^ Briefly, raw Hi‐C data were trimmed and low‐quality fragments were removed by BBmap software (version 38.16) with bbduk.sh function. HiCPro software (version 2.7)^19^ was applied to align to the mouse reference genome (mm9) using the Bowtie2 algorithm.^20^ ICE normalization was performed after filtering out uncut DNA reads, continuous reads, PCR artefacts, and so on. An interaction contact matrix was constructed by the unique mapped reads (map quality score >10) under sequential bins of 20, 40, 200, and 400 kb. The contact heatmap was visualized by the HiTC R package.^21^

### 2.5 Reproducibility of Hi‐C data

To confirm the Hi‐C data reproducibility, we calculated Pearson's correlation coefficient between two biological repeats as described previously.^10^ Briefly, we calculated the counts in each point *I* and *j* with a maximum distance restricted to 2 Mb and correlated each possible interaction *I_ij_* between two replicates. The interaction *I_ij_* was used to plot the principal component analysis (PCA) analysis.

### 2.6 Contact probability *p(s)* calculation

Contact probability *p(s)* was calculated using normalized interaction matrices at a 40‐kb resolution as described previously.^22^ Briefly, we calculate the number of interactions corresponding to distances of 40, 80, 120, and 160 kb at each interval to measure the *p(s)*. *p(s)* was then normalized according to the distances. The cooltools (version 0.4.1; https://github.com/open2c/cooltools) python package^23^ was used to calculate the locally weighted scatterplot smoothing and then plot the curve (log–log axis). A heatmap of *p*(s) was plotted by the pheatmap R package.^24^

### 2.7 Calculation of the compartment status

The HiTC^21^ package in R was applied to calculate the compartment status. Normalized matrices at 400‐kb resolution were applied to calculate the PC1 eigenvectors with the pca.hic function. Compartment strength was calculated as described previously.^25^ On the basis of PC1 values, we used the ggplot2^26^ R package to draw the compartment status distribution. Then, we used the compute‐saddle function (cooltools, version 0.4.1, https://github.com/open2c/cooltools) python package^23^ to calculate the global (genome‐wide) strength for compartmentalization as (AA + BB)/(AB + BA) after rearranging the bins of obs/exp.

### 2.8 TADs, TAD boundaries, and TAD signal calculation

TADs were called as described previously.^10^ The directional index (DI) value was calculated for each bin on the ICE normalized matrix. According to the DI value, we used a Hidden Markov Model method to call TADs. We defined TAD boundaries as <400 kb. TAD signals were calculated by the insulation score method.^27^ Briefly, to calculate the insulation score, we obtained the average number of contacts of each bin in the 20‐kb resolution by sliding a 400‐kb window. Subsequently, we fitted the insulation score with the loess method and plotted the line chart centred in the FGSC TADs (up/down to 0.5 TAD).

### 2.9 Motif identification

Enriched motifs were analysed using HOMER (v4.7)^28^ with the motif length restricted to 8, 10, and 12 nucleotides. We used the Benjamin Hochberg to adjust *p*‐value, and an applied threshold of <0.05 as the significance.

### 2.10 Identification of chromatin loops

Chromatin loops were identified as described previously.^29^ Briefly, we used the Juicer with hiccups function^30^ under the parameters ‐Xmx256g, ‐m 1024, and –ignore_sparsity. Chromatin loops with an adjusted *p*‐value of <0.001 were merged as the final identified chromatin loops.

### 2.11 Calculation of Hi‐C data of the X chromosome

To compare the Hi‐C matrix of the X chromosome during FGSC development, we followed a previously described method with a minor modification.^31^ We normalized the contacts matrix of chromosome X using the limma R package^32^ with quantile normalization and subtracted each stage of the contacts matrix of the chromosome X. The deconvolved X chromosome matrix was scaled and plotted in R.

### 2.12 RNA‐seq library generation and data analysis

An RNA library was generated as described previously.^33^ Briefly, total RNA was extracted from cells using Trizol Reagent (Invitrogen). KAPA HiFi HotStart DNA Polymerase (Roche) was used to amplify cDNA. Then, the RNA‐seq library was prepared using a Nextera XT DNA Library Preparation Kit (Illumina), following the manufacturer's instructions. The final library was amplified with phi29 (Thermo Fisher Scientific) to prepare a DNA nanoball that was loaded into a patterned nanoarray. Reads were generated on the BGISEQ500 platform. All clean paired reads were mapped to the mouse reference genome (mm9 version) using Hisat2 (version 4.8.2) with the default parameters.^34^ Then, Cufflinks (version 2.2.1) was used to measure gene expression and normalize the sequencing depth.^35^

### 2.13 Calculation of conserved domain regions

Pearson's correlation (*r*) was used to identify conserved domain regions.^36^, ^37^ Briefly, using a PC1 score matrix at a 400‐kb resolution, we slid 2‐Mb windows along the whole genome to calculate Pearson's correlation between two development stages and calculated the mean value of this correlation. Regions with *r* > 0.6 were chosen as conserved compartment regions during early development.

### 2.14 Enrichment analysis of gene ontology

Enrichment analysis of gene ontology (GO) was performed using the DAVID websites (version 6.8)^38^ with a focus on enriched terms under the biological process category. The Benjamin Hochberg to adjust the *p*‐value was used, and a *p‐*value threshold of <0.05 was applied for statistical significance.

## 3 RESULTS

### 3.1 Global chromatin structure during FGSC development

To explore the dynamics of 3D chromatin organization during FGSC development, we collected GV and MI oocytes from mouse ovaries and performed an Hi‐C^16^ experiment with two biological repetitions for each stage. We generated approximately 400 million reads for each replicate stage and obtained 140 and 343 million reads for GV and MI oocyte pairwise chromatin contacts, respectively (Table S1, Figure S1). By combining the two biological replicates of contacts matrix into a single set of merged matrix for each stage, we obtained a maximum resolution of an Hi‐C matrix at 20 kb.

**FIGURE 1:**
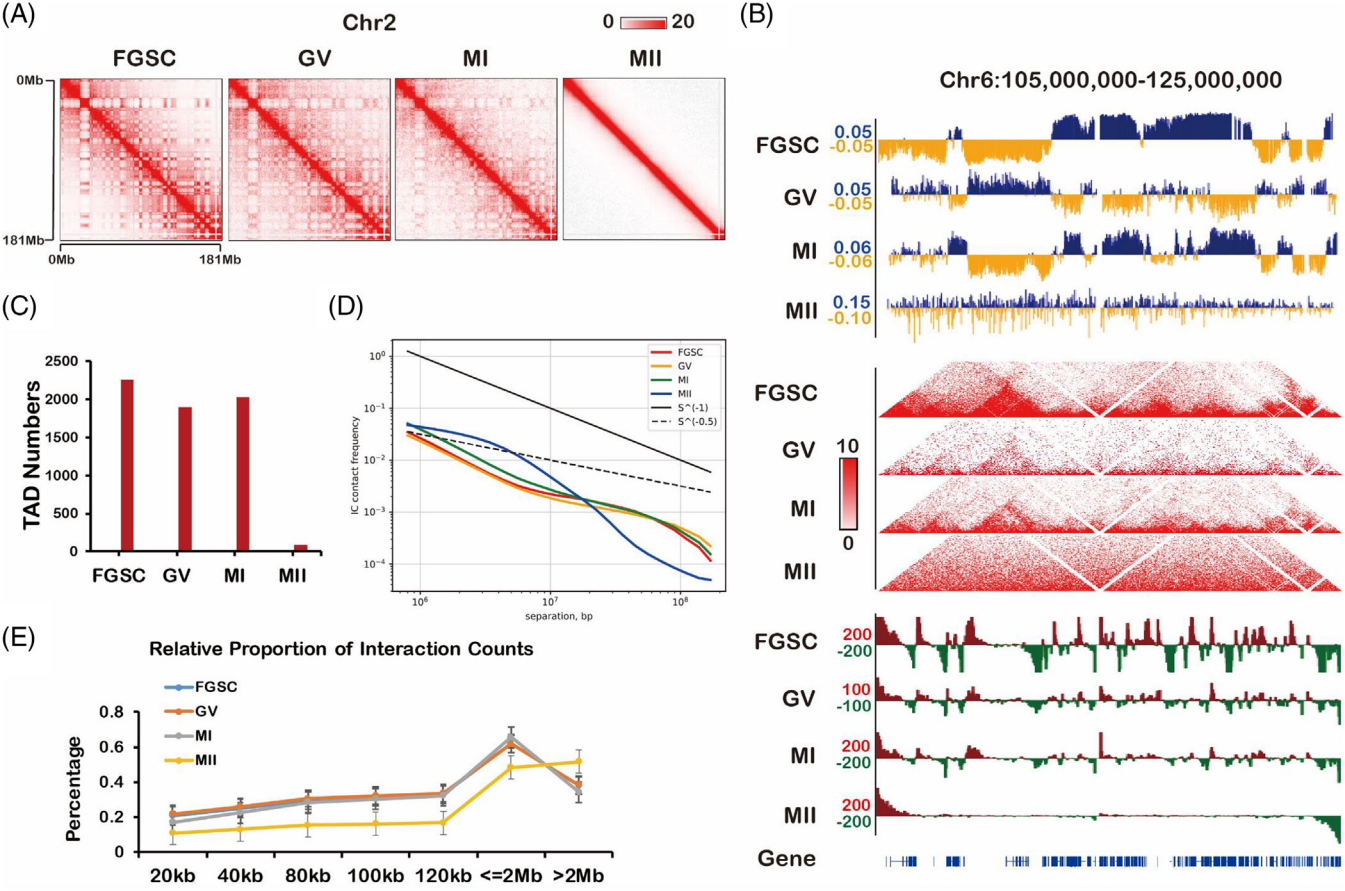
Overall chromosome organization during female germline stem‐cell (FGSC) development. (A) Contact matrices of chromosome 2 demonstrated the genome organization during FGSC development. (B) First principal component (PC1) value, normalized high‐throughput chromosome conformation capture (Hi‐C) interaction heatmaps, and directional indexes (DIs) during FGSC development at a 20‐kb resolution. PC1 values were used to indicate the A/B compartment status. Blue indicates a positive PC1 value representing the A compartment and yellow indicates a negative PC1 value representing the B compartment. (C) Numbers of identified topologically associating domains (TADs) during FGSC development. (D) Average contact probability across the genome decreased as a function of genomic distance, which demonstrated the dynamic change of contact probability during FGSC development. (E) Relative proportions of *cis* interactions at different genome distances versus total paired loci, which displayed the difference in intrachromosomal interactions during FGSC development.

We used public data of FGSCs and MII oocytes^4^, ^17^ to build a landscape of the intrachromosomal contact heatmap to show the dynamic changes in chromatin organization during FGSC development (Figure 1A, Figure S2). By avoiding sex chromosome effects, we analysed TADs that gradually disappeared during FGSC development (Figure 1B). We also found that GV and MI oocytes had similar numbers of TADs, and MII oocytes had barely detectable TADs (Figure 1C). Furthermore, by calculating the average intrachromosomal contact probability during FGSC development, it was found that the frequency of interaction contacts was reduced monotonically from 1 × 10^5^ to 1 × 10^8^ bp along the whole genome (Figure 1D). Interestingly, the contact probability of MII oocytes was significantly different from that at other stages of FGSC development, probably because of the lack of a TAD structure in the MII stage (Figure 1D). By divided *cis*‐long (>2 Mb) or *cis*‐short (<2 Mb) interactions by the total number of *cis* interactions at the whole genome, we found the relative proportion of *cis*‐short contacts in FGSCs was similar to that in GV stage oocytes and the lowest in MII stage oocytes (Figure 1E). These results suggest that high‐order chromatin organization changes dynamically during FGSC development.

### 3.2 Global 3D genome reorganization during FGSC development

**FIGURE 2:**
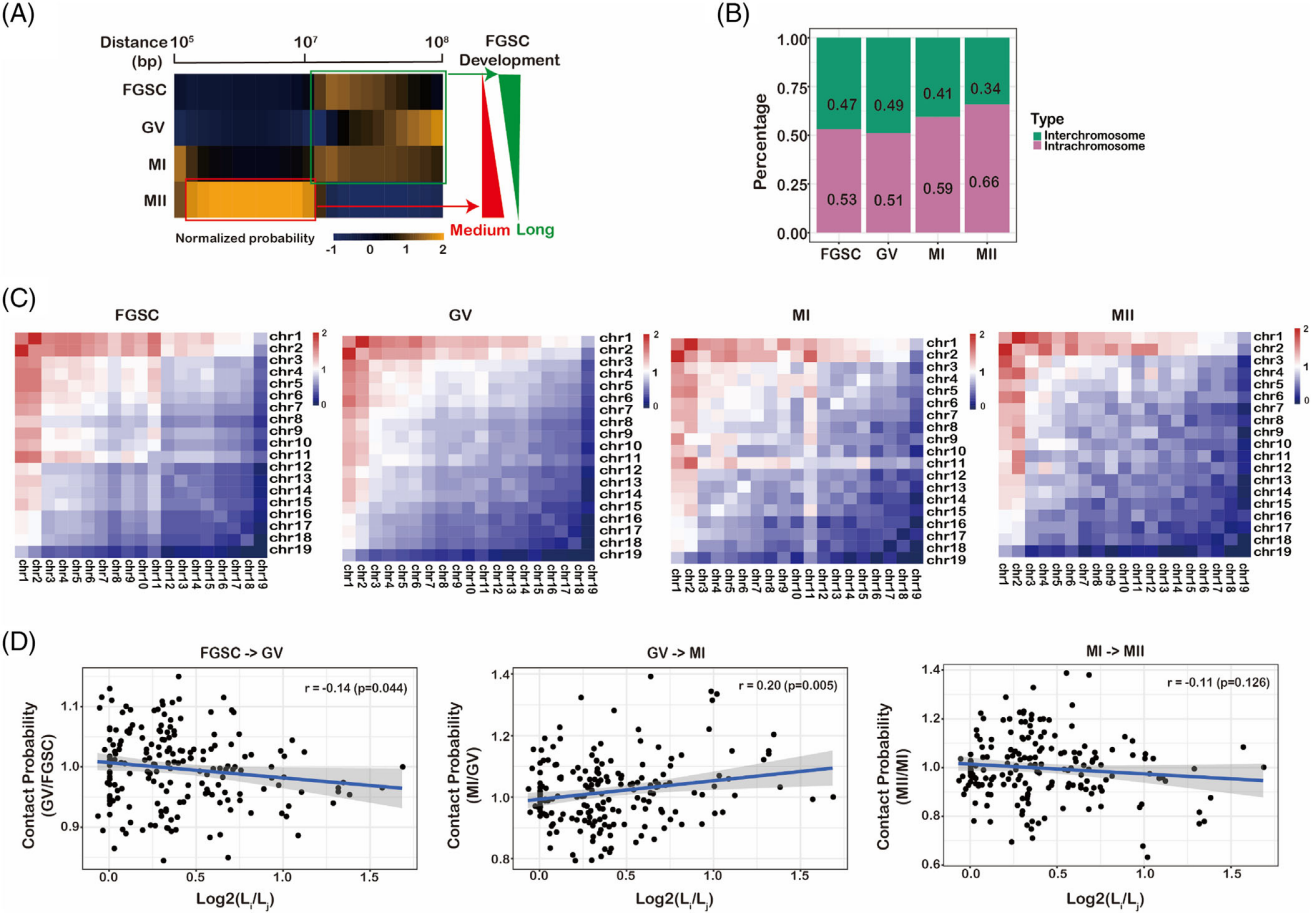
Global 3D genome reorganization during female germline stem‐cell (FGSC) development. (A) Heatmap of the normalized contact probability relative to genomic distance, which showed reorganization of the 3D genome structure during FGSC development. Red, medium‐distance interactions; green, long‐distance interactions. (B) Percentage of interchromosomal and intrachromosomal contacts indicated the global change of contacts during FGSC development. (C) Observed/expected number of contacts between pairs of autosomes, which displayed the different *trans* contacts during FGSC development. (D) Observed/expected number of interactions between autosomes with normalization of the chromosome lengths. The length difference was calculated as log_2_ *L_i_*/*L_j_*, *L_i_* > *L_j_*, where *L_i_* and *L_j_* represent the lengths of two chromosomes. The dotted line indicates the trend suggesting a relationship between chromosome length and contact frequency.

To understand spatiotemporal chromatin structure during FGSC development from a global viewpoint, we analysed the contact probability dependence on the genomic distance at each stage of FGSC development. We found that the medium‐distance interactions from 1 × 10^6^ to 1 × 10^7^ bp were enriched in MII oocytes, whereas long‐distance interactions from 1 × 10^7^ to 1 × 10^8^ bp were enriched at the other stages (Figure 2A). We compared the percentages of long‐distance interactions between interchromosomal and intrachromosomal contacts and found that interchromosomal contacts were decreased, whereas intrachromosomal contacts were increased for all chromosomes during FGSC development (Figure 2B, Figure S3), suggesting that FGSC development was accompanied by drastic remodelling of chromosomal territories. By applying the observed/expected number of contacts between pairs of autosomes, we found that only chromosomes 1 and 2 in the MII stage had high interactions with other chromosomes, and preferential contacts between chromosomes 1 and 11 were the most frequent in the other stages (Figure 2C). By comparing the contact probability of each chromosome in accordance with its length, we found that contacts among long and short chromosomes decreased preferentially during FGSC–GV oocyte transition and increased during GV–MI oocyte transition. However, we found no significant difference in contact probability during MI–MII oocyte transition (Figure 2D). These results suggest that the global 3D genome reorganizes dramatically during FGSC development.

### 3.3 Dynamic chromatin compartmentalization during FGSC development

**FIGURE 3:**
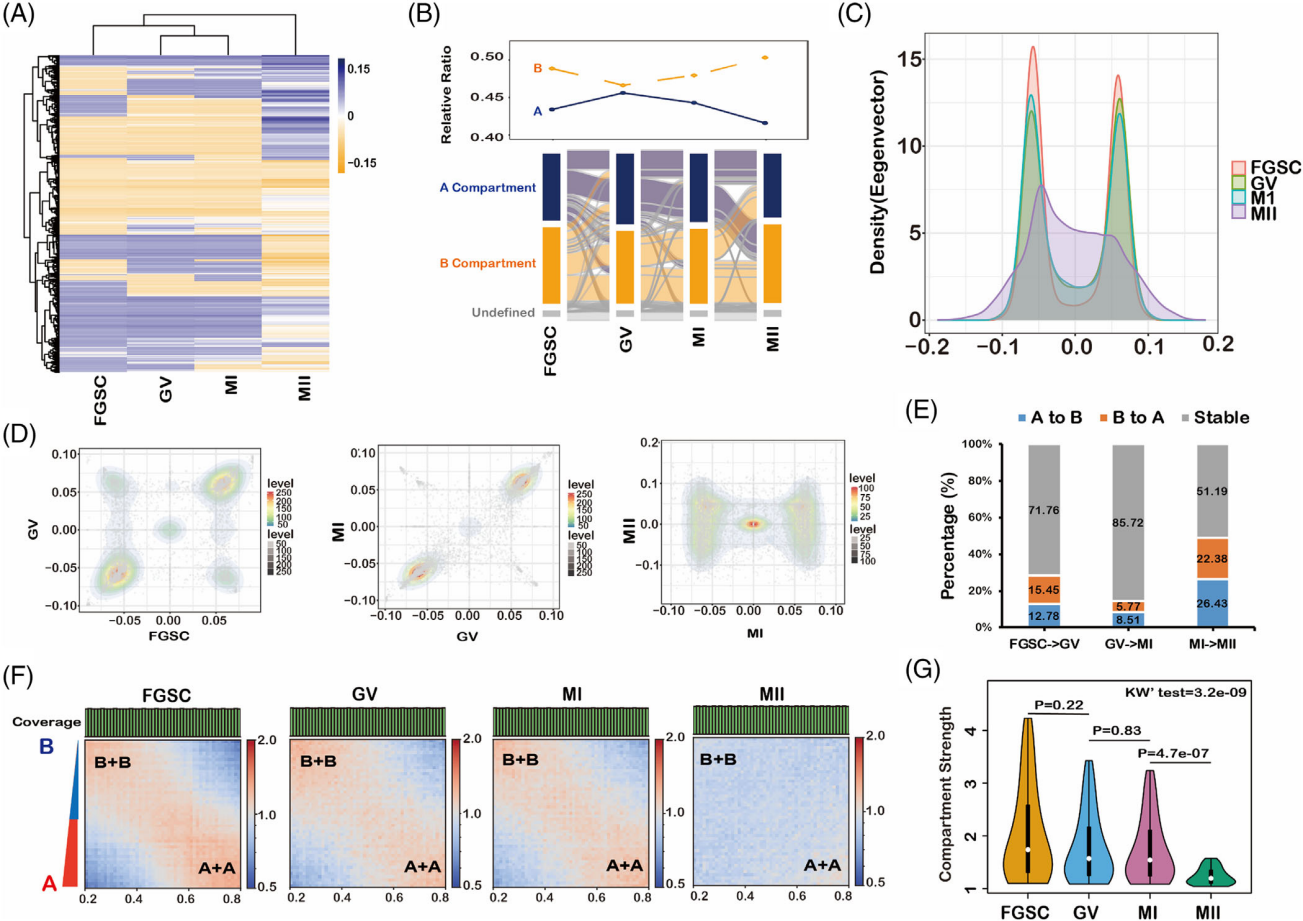
Dynamic chromatin compartmentalization during female germline stem‐cell (FGSC) development. (A) Heatmap of the change in compartment status during FGSC development. (B) Sankey plot showing the dynamic switch of the A/B compartment status during FGSC development. (C) Distribution of eigenvector values considering autosomes, which demonstrated the PC1 score distribution during FGSC development. (D) Scatter density plot of the compartment status distribution during FGSC development. (E) Percentage of switched A/B compartments occupied in the genome during FGSC development. (F) Saddle plots showing that the (A + A) and (B + B) compartment strengths were dynamically changed during FGSC development. (G) Violin plot of compartment strength, which showed a decrease during FGSC development (*p*‐value calculated by Wilcoxon's test).

To further explore changes in the chromosome organization during FGSC development, we determined the A/B compartment status by hierarchical analysis. The compartment status of GV oocytes was similar to that of MI oocytes and different from that of FGSCs and MII oocytes (Figure 3A, Figure S4). RNA‐seq result displayed that genes had higher expression in compartment A than in compartment B (Figure S5) during FGSC development, which is consistent with a previous study.^14^ The Sankey plot demonstrated that the proportion of the A compartment decreased and the B compartment increased gradually during FGSC development (Figure 3B). Interestingly, GV oocytes had the highest ratio of A compartments and lowest ratio of B compartments, probably because of the distinctiveness of the first meiosis stage. Furthermore, in principal component analysis, most of the first principal (PC1) scores were well distributed in (A + A) and (B + B) quadrants for FGSC–GV oocyte and GV–MI oocyte transitions, which was different from the PC1 scores for the MI–MII oocyte transition, suggesting that the compartment status switches greatly during FGSC development (Figure 3C,D). We found that 50% of A/B compartments had switched in the MI–MII oocyte transition, whereas approximately 70%–80% of the compartments were stable during FGSC–GV oocyte and GV–MI oocyte transitions (Figure 3E). The compartment strengths in FGSCs were similar to those in GV stage oocytes and significantly decreased in MII stage oocytes (Figure 3F,G). These results not only demonstrate that compartments exist and weaken during FGSC development, but also that the compartments switch greatly during MI–MII oocyte transition.

### 3.4 TADs attenuate and then disappear during FGSC development

**FIGURE 4:**
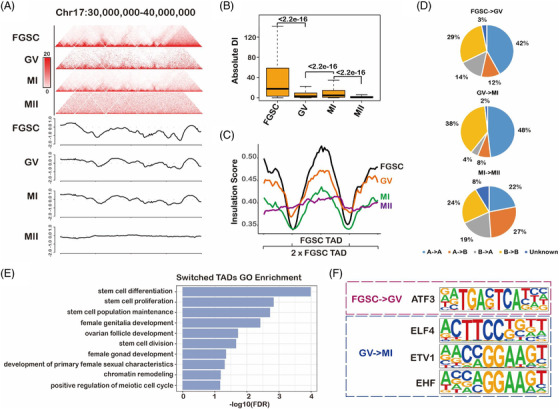
Topologically associating domains (TADs) attenuate and then disappear during female germline stem‐cell (FGSC) development. (A) Normalized high‐throughput chromosome conformation capture (Hi‐C) interaction frequencies displayed as a heatmap and TAD signals during FGSC development at chromosome 17: 30–40 Mb. (B) Boxplot of absolute directional index (DI) values, which demonstrated a decrease in the DI score during FGSC development (*p*‐value calculated by the Kruskal–Wallis test). (C) Average insulation scores (IS) of different stages, which showed a decrease in the TAD strength during FGSC development at TADs (defined in FGSCs) and nearby regions. (D) Percentage of switched TADs in the genome during FGSC development. (E) Gene Ontology enrichment analysis of genes with promoters located in switched TADs during FGSC development. (F) Enriched motifs in switched TADs during FGSC development, which indicated that the genes may contribute to FGSC development.

To explore the dynamic changes in TADs during FGSC development, we first visualized the normalized contact maps at chr17:30–40 M and found weakening of the TAD strength during FGSC development (Figure 4A). It was in contrast to the changes reported in TADs during spermatogenesis.^39^ To further systematically identify the changes in TADs during FGSC development, we calculated the degree of interactions of 20‐kb bins in upstream or downstream regions (≤2 Mb) as the DI value, which could measure the TADs strength. Expectedly, the DI value was significantly decreased during FGSC development (Figure 4B). Furthermore, we measured the TAD boundary strength with the insulation score, the result illustrated that the boundaries were strongest in FGSCs, reduced in GV and MI stage oocytes, and weakest in MII stage oocytes (Figure 4C). It indicates that TADs attenuate in GV and MI stage oocytes and disappear in MII stage oocytes.

To identify dynamic changes in TAD organization, we evaluated the TAD distribution in A and B compartments in accordance with the PC1 scores. We found that the percentage of A‐ to B‐type TADs was increased during MI–MII oocyte transition at the genome‐wide level during FGSC development as well as the B‐ to A‐type of TADs (Figure 4D). To determine whether the switch of TADs was biologically meaningful, we performed RNA‐seq. We first identified 7412, 6605, and 5182 significantly differentially expressed genes in FGSC–GV, GV–MI, and MI–MII transitions, respectively (Figure S6). Next, we found that the TAD switch was accompanied by significant changes in gene expression in FGSC–GV transition (Figure S7A). We further conducted GO functional enrichment of genes with promoters located in the switched TADs. The results demonstrated that these genes were related with ovarian development, stem‐cell development, chromatin remodelling, and the meiotic cell cycle (Figure 4E, Table S2), which is consistent with our prior findings of FGSC development.^6^, ^7^, ^8^ Moreover, motif enrichment of the switched TAD regions indicated that activating transcription factor 3 (ATF3) might play a role in FGSC–GV oocyte transition, whereas E74‐like ETS transcription factor 4 (ELF4), ETS‐variant transcription factor (ETV1), and ETS homologous factor (EHF) may be important for GV–MI oocyte transition (Figure 4F). RNA‐seq showed that ATF3 and ELF4 were the most highly expressed in FGSCs, suggesting that ATF3 has an important role in FGSC development (Figure S7B).

Next, to further understand the potential mechanism of ATF3 in FGSC development, we analysed chromatin loops during FGSC development. We first found a reduction in chromatin loops during FGSC development, which identified 3332, 579, 209, and 55 chromatin loops in FGSCs, and GV, MI, and MII oocytes, respectively. Combined with the RNA‐seq data, we found that genes were more highly expressed within loops than out of loops (Figure S8A), suggesting that chromatin loops correlated with gene expression. Unexpectedly, the genes were inactivated during FGSC development (Figure S8B), which was consistent with the decrease of chromatin loops. Next, we found that the FOS‐like 2 (Fosl2) promoter had formed a chromatin loop in FGSCs with the highest expression, which loosened in GV, MI, and MII oocytes (Figure S8C). ATF3 and Fosl2 both belong to the AP‐1 family in which they have an interaction, in accordance with a previous report.^40^ Fosl2 is related to female gonad development.^41^ The results indicated that ATF3 may act as a transcription factor mediating the formation of the chromatin loop to activate the expression of Fosl2 to regulate FGSC development. Overall, our results show that TADs attenuate and then disappear during FGSC development, and their dynamic switching contributes to FGSC development.

### 3.5 X chromosome organization during FGSC development

**FIGURE 5:**
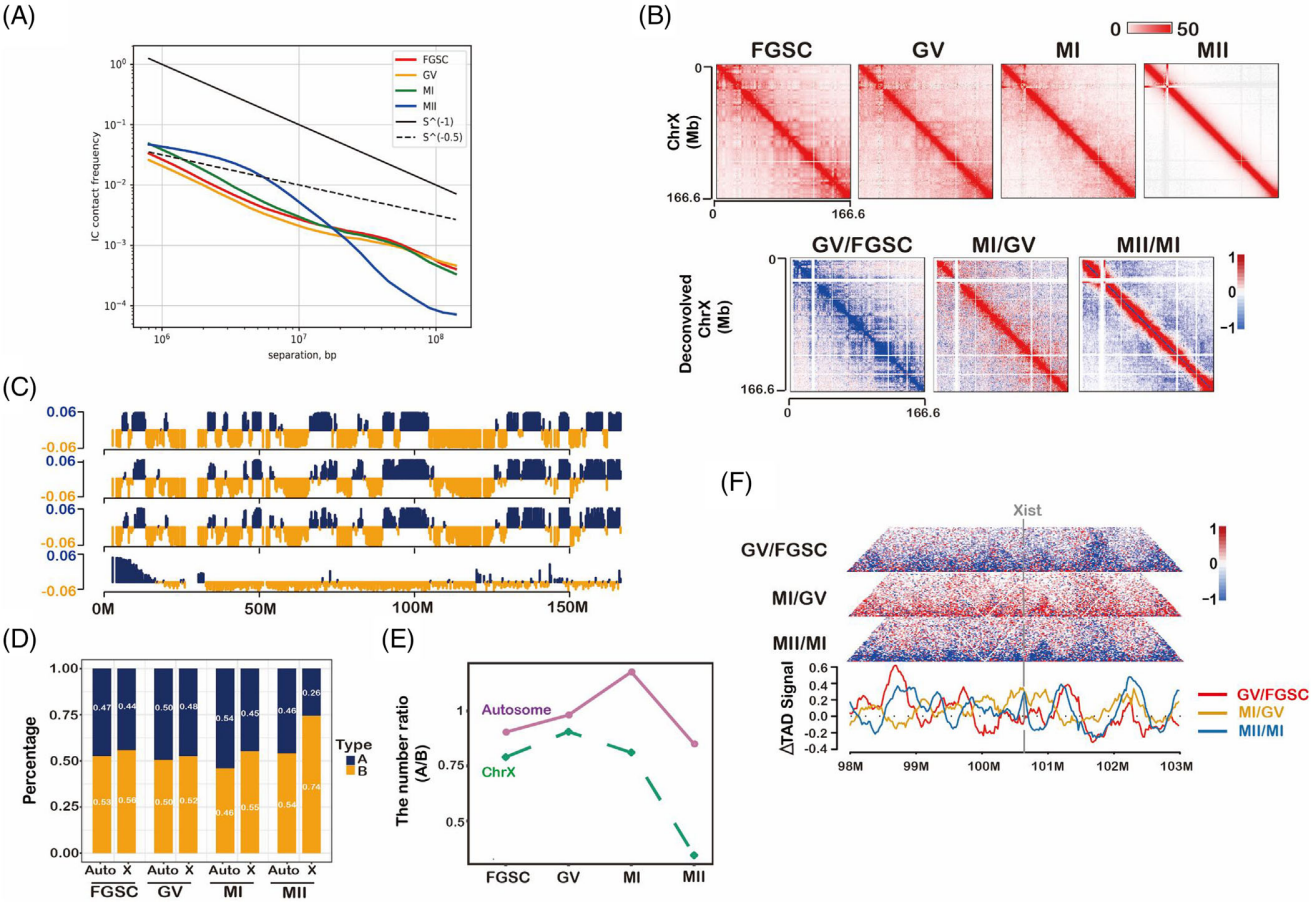
X chromosome organization during female germline stem‐cell (FGSC) development. (A) *p(s)* curve displaying the difference in average contact probability across the X chromosome during FGSC development. (B) Contact heatmap of the X chromosome (upper) and deconvolution of high‐throughput chromosome conformation capture (Hi‐C) data by subtracting each stage‐normalized matrix during FGSC development (lower). (C) PC1 score of the X chromosome during FGSC development. (D) Percentage of the compartment status, which showed the difference in compartment status between autosomes and the X chromosome during FGSC development. (E) Number ratio of A/B compartments, which demonstrated the compartment status switch in autosomes and the X chromosome during FGSC development. (F) Normalized deconvolved chromatin interaction maps of the *Xist* region at a 20‐kb resolution with topologically associating domain (TAD) signals during FGSC development.

Considering that the X chromosome undergoes inactivation–activation during FGSC development, we examined the chromatin architecture of the X chromosome during FGSC development. The contact probability of the X chromosome was different from that of autosomes, particularly medium‐distance intrachromosomal contacts (1 × 10^6^–10^7^ bp) (Figure 5A), suggesting a change in chromosome organization during intrachromosomal interactions. A heatmap of X chromosome contacts at each stage showed a dramatic reduction in contacts during FGSC–GV oocyte transition and an increase during GV–MI oocyte transition, and a strong increase for short‐distance contacts and a decrease for long‐distance contacts during MI–MII oocytes transition (Figure 5B). To further understand the differences in chromosome organization between the X chromosome and autosomes, we first confirmed the compartment status of the X chromosome during FGSC development, which was consistent with that of autosomes (Figure 5C). The percentage of the A compartment was higher in autosomes than that in chromosome X (Figure 5D), and the number of A/B compartments in autosomes and the X chromosome showed different trends (Figure 5E). Specifically, the ratio of the number of A/B compartments was decreased in the X chromosome and increased in autosomes during FGSC development, suggesting that the X chromosome and autosomes had different chromosome organizations. Additionally, we assessed the TAD signal to measure organization in the *Xist* region. The results showed that *Xist* was not located in the valley bottom of TAD signals, suggesting that *Xist* transcription was not activated during FGSC development (Figure 5F), consisting with a previous report that the X chromosome activates in GV oocytes.^42^

### 3.6 Comparison of high‐order chromatin organization between female and male germline development

**FIGURE 6:**
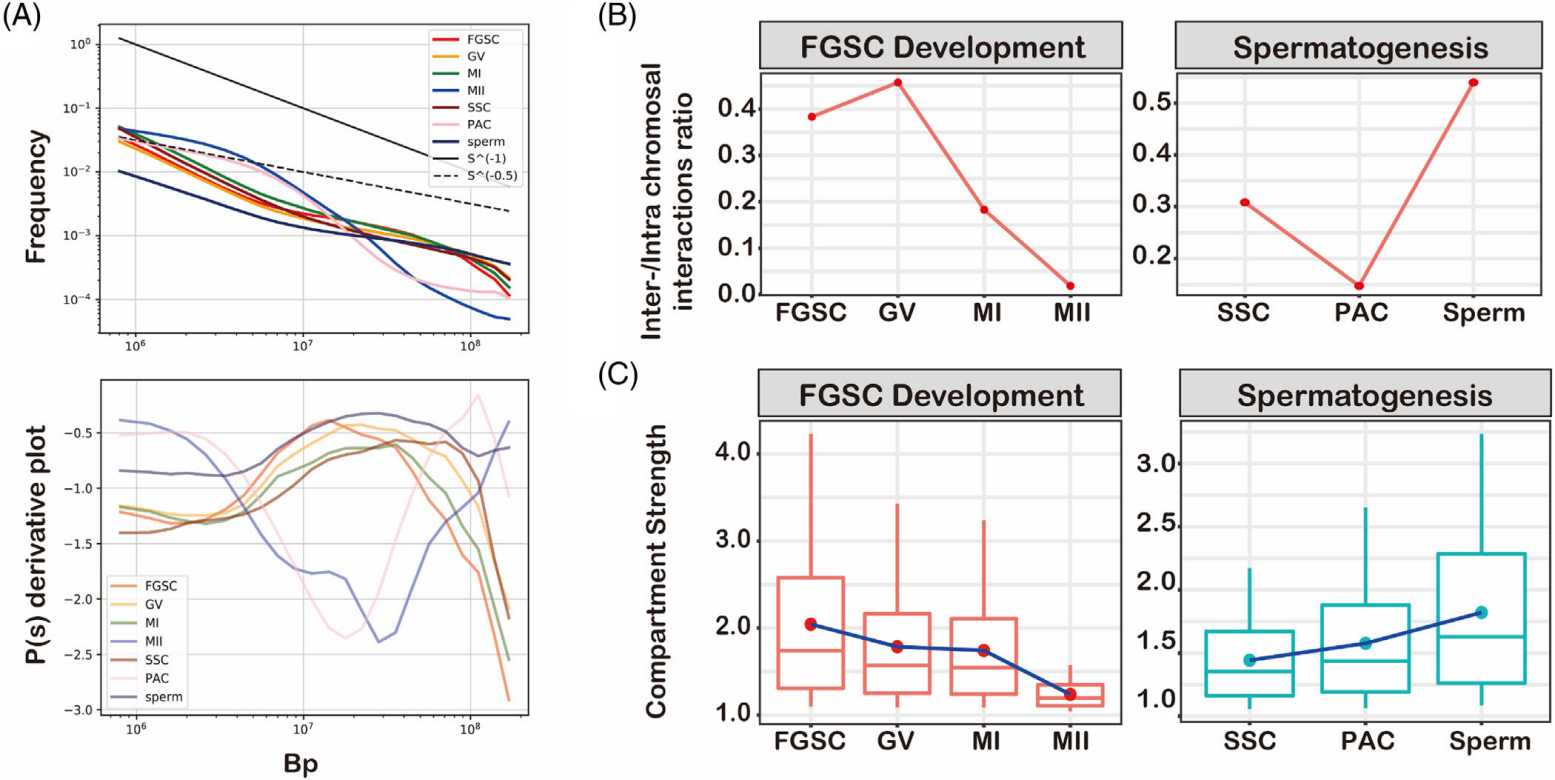
Comparison of three‐dimensional (3D) genomes between female and male germline development. (A) *p(s)* curve demonstrating the difference in average contact probability across the genome, which decreased as a function of genomic distance during gamete development. Top: *p(s)* curve during gamete development; Bottom: *p(s)* derivate slope curve during gamete development. (B) Line chart of the two different patterns of interchromosomal and intrachromosomal interaction ratios between female germline stem cell (FGSC) development and spermatogenesis. (C) Boxplot of the difference in compartment strengths for A–A and B–B interactions between FGSC development and spermatogenesis.

**FIGURE 7:**
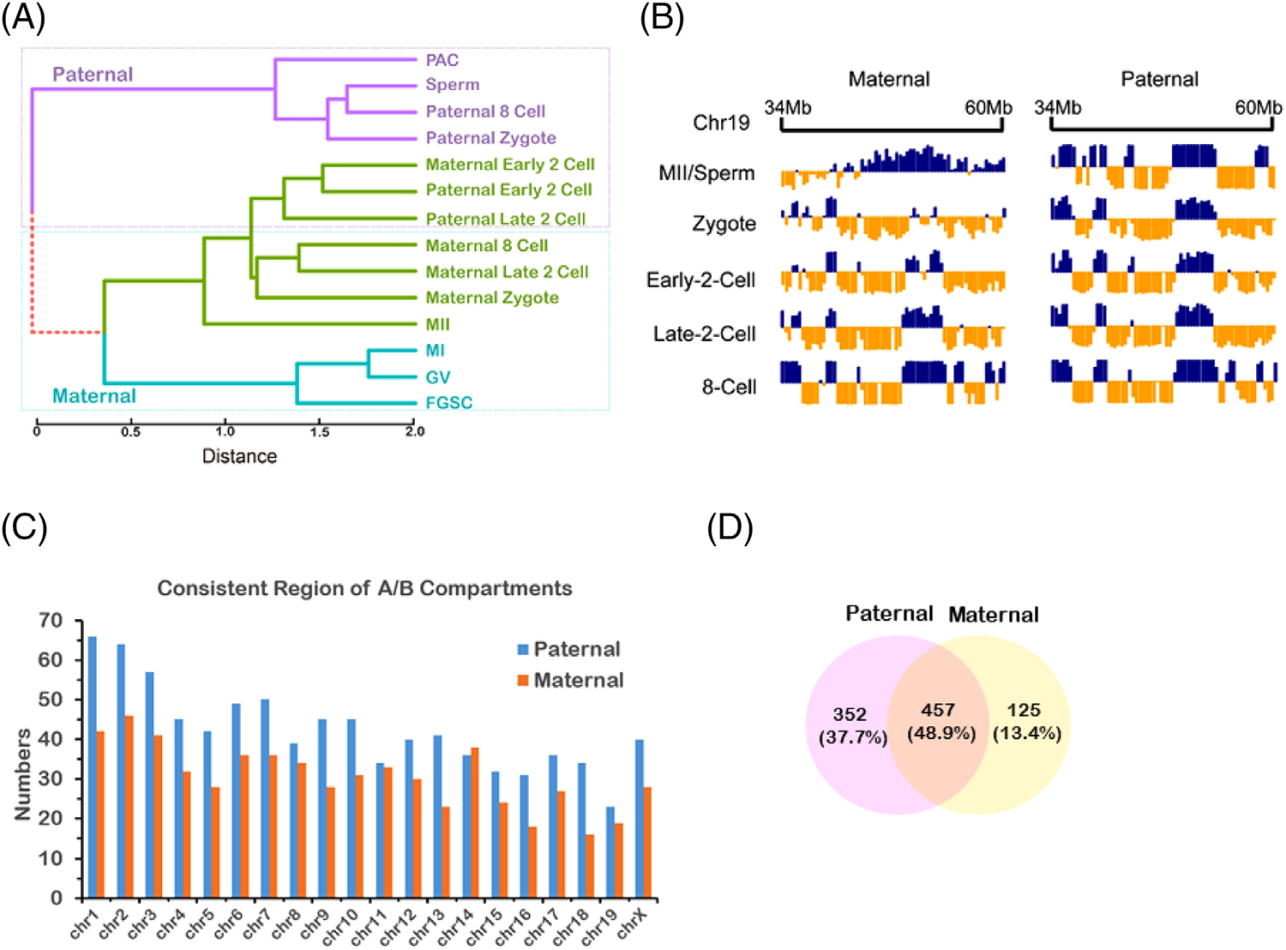
Identification of conserved allelic chromatin structures. (A) Hierarchical clustering of PC1 values on the basis of maternal (black) and paternal (red) genome architectures, which indicated conservation of the allelic chromatin structure in early embryonic development. The reference genome is shown in green. (B) Example of the conserved region at chromosome 19: 34–60 Mb during early embryonic development. (C) Number of conserved regions was higher in the paternal genome than the maternal genome. (D) Venn diagram showing half of the conserved regions in paternal and maternal genomes was overlapped.

Although both female and male germline developments undergo two rounds of meiosis accompanied by dramatic remodelling of chromatin organization, the difference in the 3D chromatin structure during gamete development remains unknown. We first assessed overall changes in chromatin organization during meiosis by distance‐dependent interaction frequencies, including FGSC development and spermatogenesis.^4^, ^5^ We found that the power‐law with the slope of contact probability showed a concave curve in MII oocytes and PACs, exhibiting the lowest slope of ∼−2.3 at a genomic distance of 11 Mb (Figure 6A). However, the slope showed a convex curve among other stages of meiosis with the highest slope of ∼−0.5 at a genomic distance of 11 Mb (Figure 7A). This suggested that MII oocytes and PACs had remodelled more drastically than other stages of meiosis in terms of the 3D chromatin organization. The opposite trend of the slope between MII oocytes and sperm indicated two different molecular mechanisms of chromatin reorganization between FGSC development and spermatogenesis. Furthermore, we compared interchromosomal and intrachromosomal interactions during gametes development. The results showed an increase of interchromosomal interactions in GV oocytes and then a decrease in MI and MII oocytes (Figure 6B). However, it was decreased at PACs and then increased at sperm, which was the opposite trend to FGSC development. Next, we evaluated the compartment strength based on the A–A and B–B intrachromosomal interactions. The results showed the opposite trend between female and male germline development, which decreased during FGSC development, but increased during spermatogenesis (Figure 6C). By counting the number of TADs in female and male germline developments, we found the TAD was decreased in PACs and recovered in sperm, whereas it was gradually attenuated in MI oocytes and then disappeared in MII oocytes (Figure S9). Taken together, these data showed remodelling of the 3D genome organization by two different potential mechanisms to package the chromatin during gamete development, in which interchromosomal interactions, compartments, and TADs were decreased during FGSC development, but reorganized and recovered during spermatogenesis.

### 3.7 Identification of conserved allelic chromatin structures

At the last stage of FGSC development, MII oocytes fuse with sperm to form zygotes. To explore the chromosome organization in MII oocytes and zygotes, the published Hi‐C data of early embryonic cells from two mouse strains were analysed.^4^, ^5^ Based on these data, we can track maternal and paternal genomes by using the single nucleotide polymorphisms. By hierarchical clustering of PC1 score in accordance with the compartment status, we found that the paternal genome was clustered in paternal zygotes, eight‐cell embryos, PACs, and sperm, whereas the maternal genome was clustered in maternal zygotes, eight‐cell embryos, GV, MI, and MII oocytes (Figure 7A), suggesting the conservation of the maternal and paternal genome in early embryonic development. At the early two‐cell‐stage embryos, the maternal genome was clustered with the paternal genome, whereas it was completely separated at the later stages. These results indicated that the high‐order chromatin architecture reorganized drastically in the early two‐cell‐stage embryos, possibly because of TAD re‐establishment at this stage. Moreover, we found that some regions based on the PC1 score were conserved in maternal and paternal genome during early embryonic development (Figure 7B). To systematically identify the conserved regions, we calculated Pearson's correlation that occurred across each 2‐Mb sliding window between maternal and paternal genomes. The result showed that the higher number of conserved regions in paternal genome than in the maternal genome (Figure 7C,D), implying that the paternal genome was more conserved. By enriching those allelic‐specific regions with imprinted genes, we found that most of the imprinted genes were significantly related to the conserved regions (Table S3) (Fisher's exact test, adjusted *p* by Benjamin Hochberg, *p* < 0.05). These regions contained insulin‐like growth factor 2 receptor(*Igf2r*), delta‐like non‐canonical Notch ligand 1 (*Dlk1*), and iodothyronine deiodinase 3 (*Dio3*) genes that are highly expressed in maternal or paternal genomes.^43^, ^44^ The results suggest that the allelic‐specific conserved region affects these imprinted genes, which may regulate paternal or maternal development.

## 4 DISCUSSION

The chromatin architecture is essential in germline cells because it carries the necessary information to pass on to the next generation. FGSCs undergo two meiosis events with drastic chromatin reorganization to become mature oocytes. However, little is known about the high‐order chromatin structure during FGSC development. Here, we examined the dynamics of the chromatin structure at each stage of FGSC development in mice. We found that MII oocytes had the lowest proportion of interchromosomal contacts, implying that the chromosome territory and karyotype become more clearly structured during FGSC development. Interestingly, GV oocytes had a similar compartment status to MI oocytes, probably because GV and MI oocytes are undergoing the meiosis phase. Conversely, the compartment changed dramatically during MI–MII oocyte transition, possibly because the polar body forms in MII oocytes. This possibility raises the issue of whether the polar body and chromatin organization are related. We next found that TADs were reduced and then vanished during FGSC development. The disappearance of TADs in MII oocytes has been reported previously.^45^, ^46^ However, we found that TADs were weakend in GV oocytes and still present in MI oocytes, but had disappeared in MII oocytes. This finding suggests that meiosis itself is not the reason that TADs disappear and rather their disappearance in MII oocytes may be caused by polar body formation. Moreover, we found that the switch between TADs was related to their biological processes during FGSC development, in which ATF3 might play an important role during FGSC–GV oocyte transition, and ELF4, ETV1, and EHF play roles during GV–MI oocyte transition. The recent finding that ATF3 contributes to female development supports this result.^47^

FGSCs have an inactivated X chromosome that activates at the GV stage.^42^ However, the organization of X chromatin during FGSC development has not been reported until now. Our data not only revealed the chromatin structure dynamics of the X chromosome during FGSC development but also showed differences in the chromatin architecture between the X chromosome and autosomes during FGSC development. The X chromosome occupied a smaller proportion of the A compartment than autosomes, leading to a reduction in the proportion of the A compartment occupied by the X chromosome during FGSC development. These findings provide information about the X chromosome structure, which can be applied to subsequent research.

MII oocytes, as a source of maternal genetic material, fuse with sperm to form zygotes. We investigated the relationship between the chromatin organization during FGSC and early embryonic development. At the early two‐cell‐stage embryos, the maternal genome clustered with the paternal genome, while it was completely separated at the later stages, which suggested the conservation of chromatin structure in FGSC and early embryonic development. Furthermore, the conserved structures were widely present in the genome during early embryonic development.

In summary, we present a comprehensive landscape of the high‐order genome organization during FGSC development. Our findings describe the dynamic features of the chromatin organization during FGSC development, including chromatin packaging, A/B compartment switching, and TADs, which may contribute to understanding the biological processes of FGSC development. These data provide a valuable resource for future research on the molecular mechanism of genome organization during FGSC development and have important implications for medical study and potential and actual clinical applications.

## CONFLICT OF INTEREST

The authors have declared that no conflict of interest.

## Supporting information

Supporting Information

Supporting Information

Supporting Information

Supporting Information
