# Supplementary material for: Three‐dimensional genome structure shapes the recombination landscape of chromatin features during female germline stem cell development
Source: Clin Transl Med. 2022 Jun 22;12(6):e927. doi: 10.1002/ctm2.927 (PMC9214757; doi:10.1002/ctm2.927)
Supplement: Supplementary file 1 — Supporting Information [file CTM2-12-e927-s001.pdf]

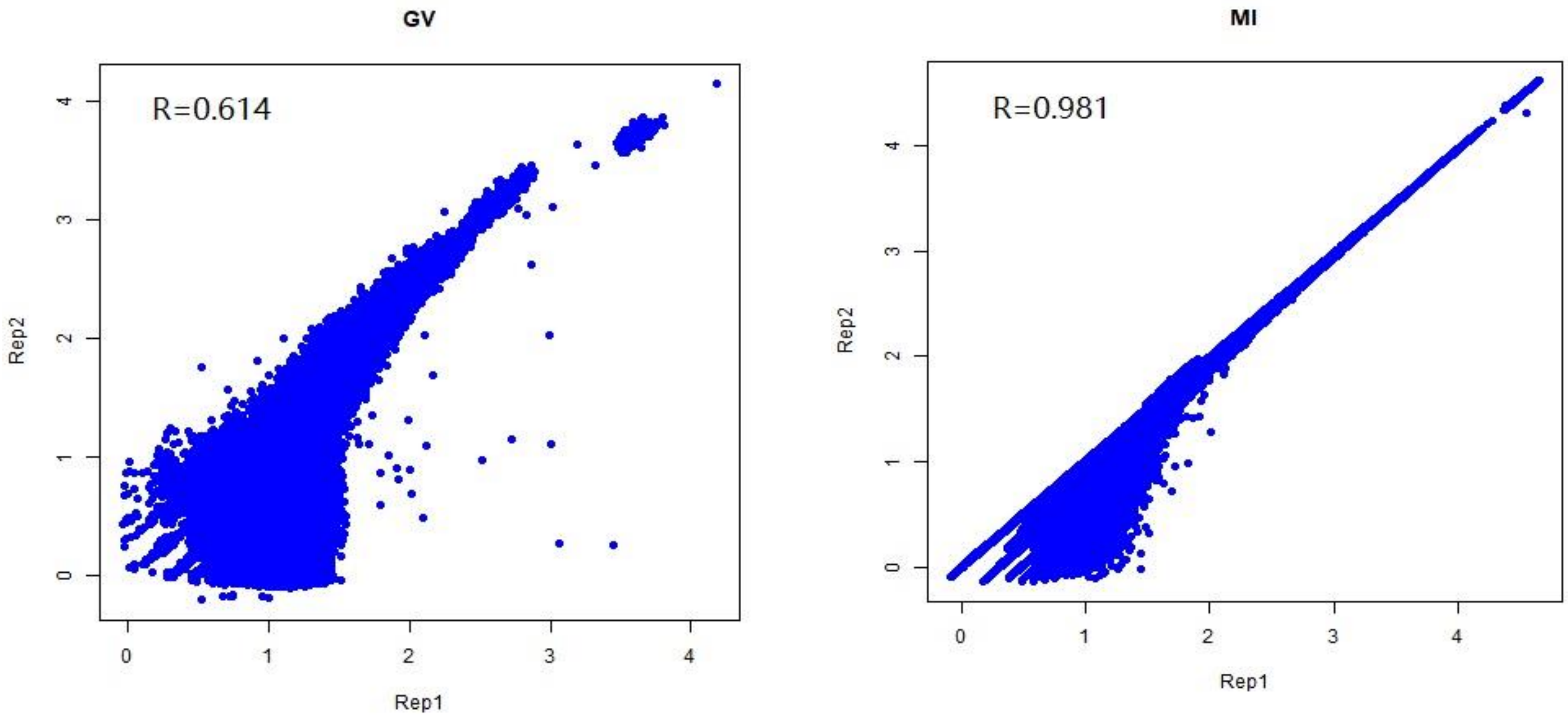

Figure S1. Validation of Hi-C data quality.

According to the normalized interaction frequency, the Pearson's correlation between Hi-C replicates for each GV and MI.

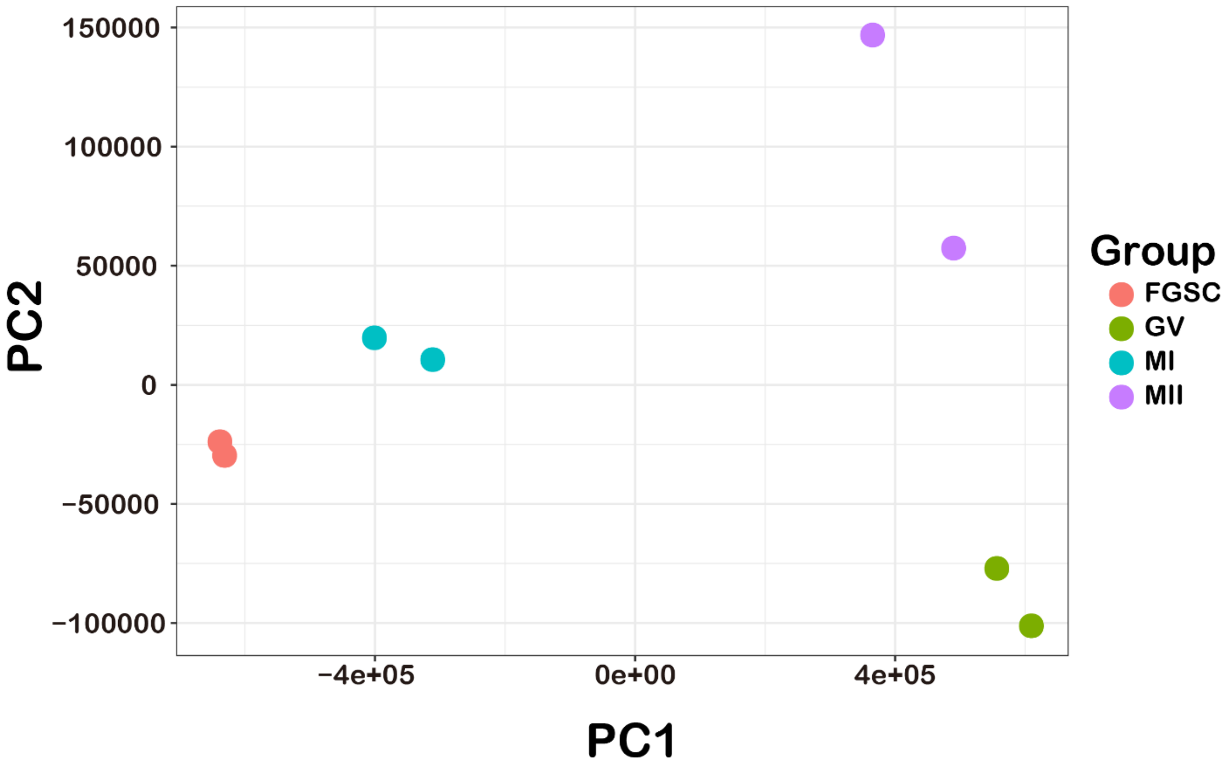

Figure S2. Validation of Hi-C data for each biological replicates.

PCA showed each stage of Hi-C data were different and each biological replicates were consistent.

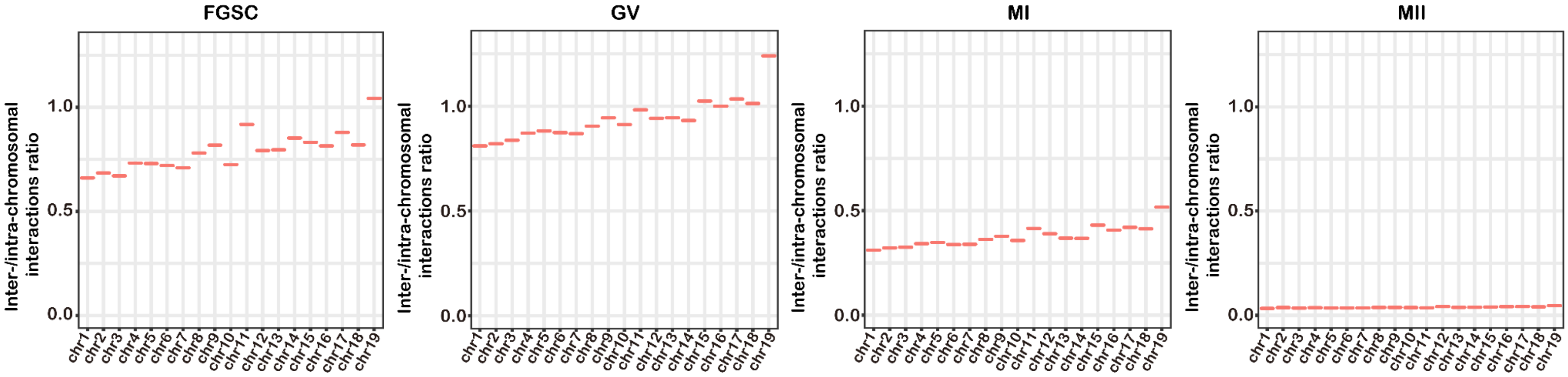

Figure S3. **Inter-/intra-chromosomal interactions ratio during FGSC development.**

Interchromosomal and intrachromosomal interaction ratios for each autosome during FGSC development.

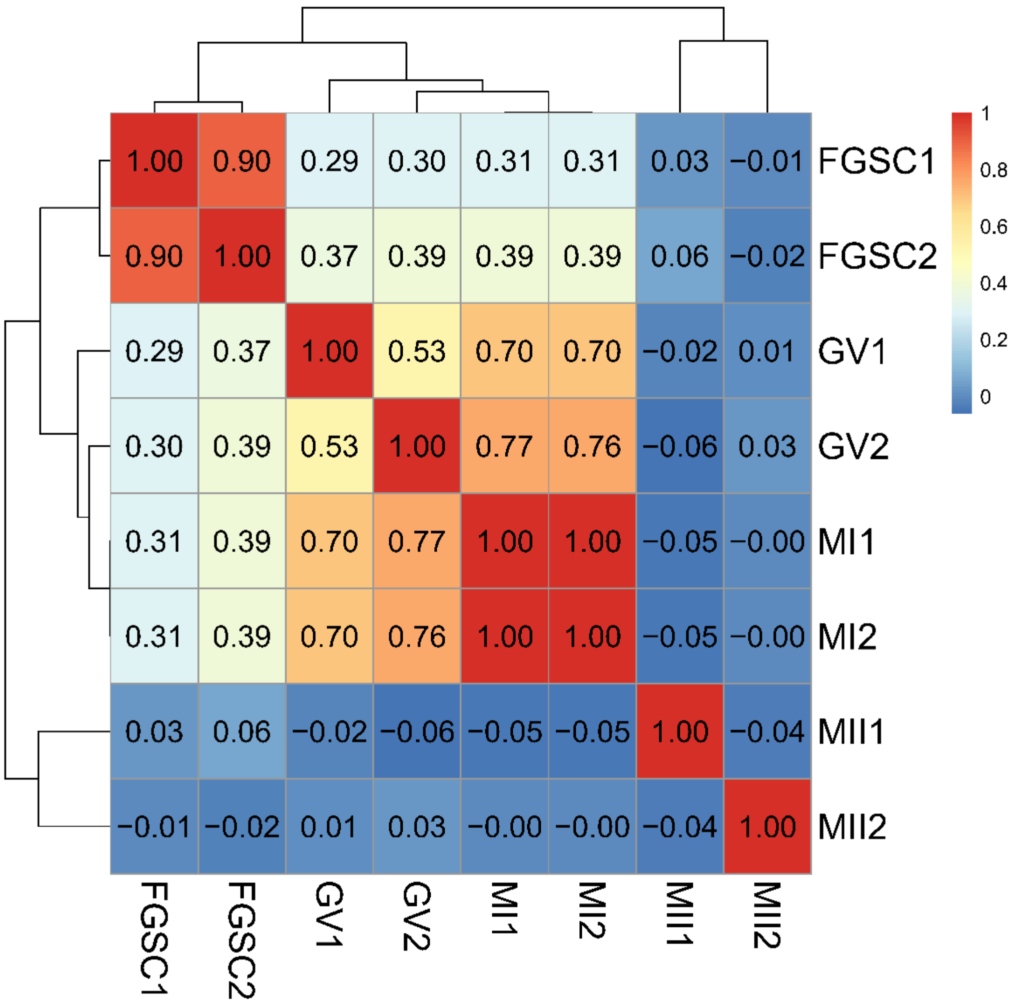

Figure S4. **Correlation of PC1 score among biological replicates**

Heatmap showed the correlation of compartment among biological replicates based on PC1 score during FGSC development.

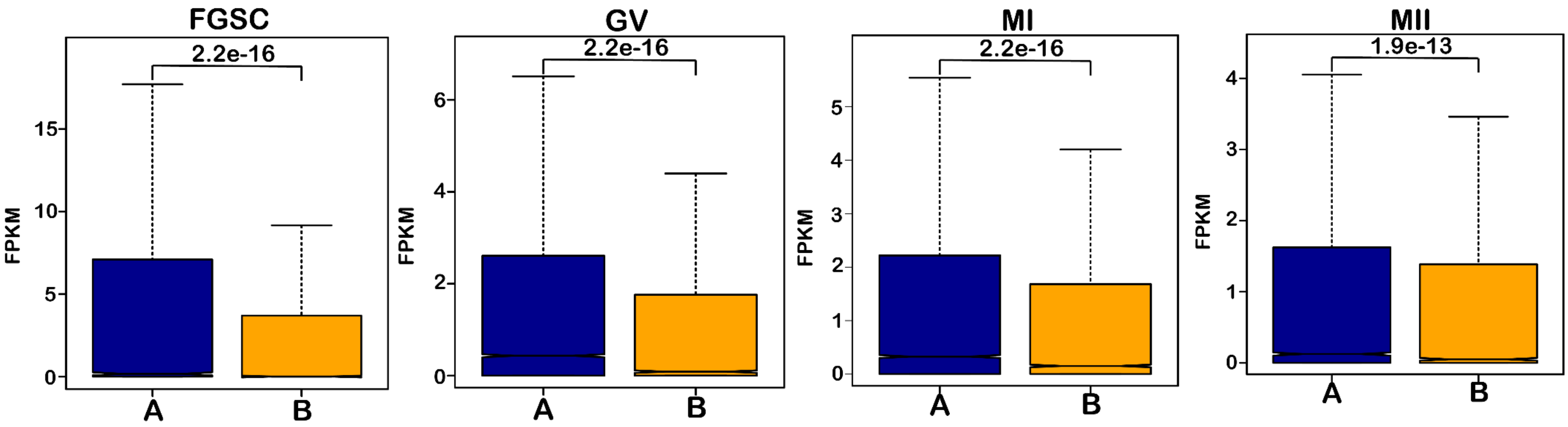

Figure S5. Gene expression of A or B compartment during FGSC development

Genes were higher expressed in A compartment than in B compartment during FGSC development.

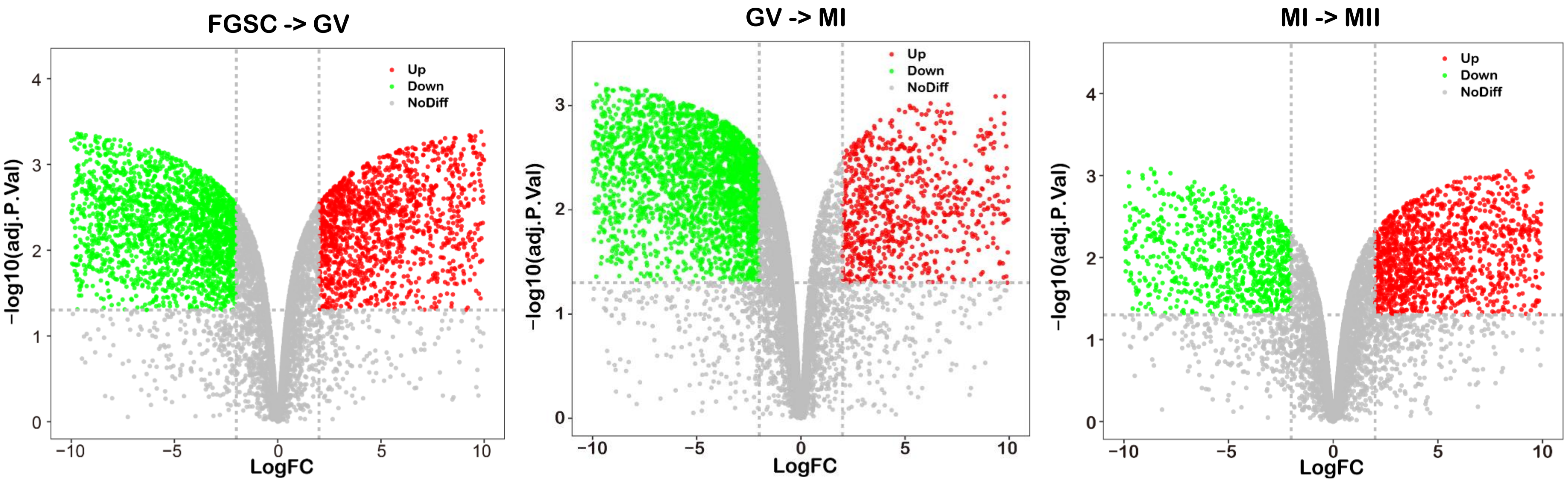

Figure S6. Dynamic gene expression during FGSC development

Volcano plot showed the significant different expressed genes in FGSC to GV, GV to MI and MI to MII, respectively.

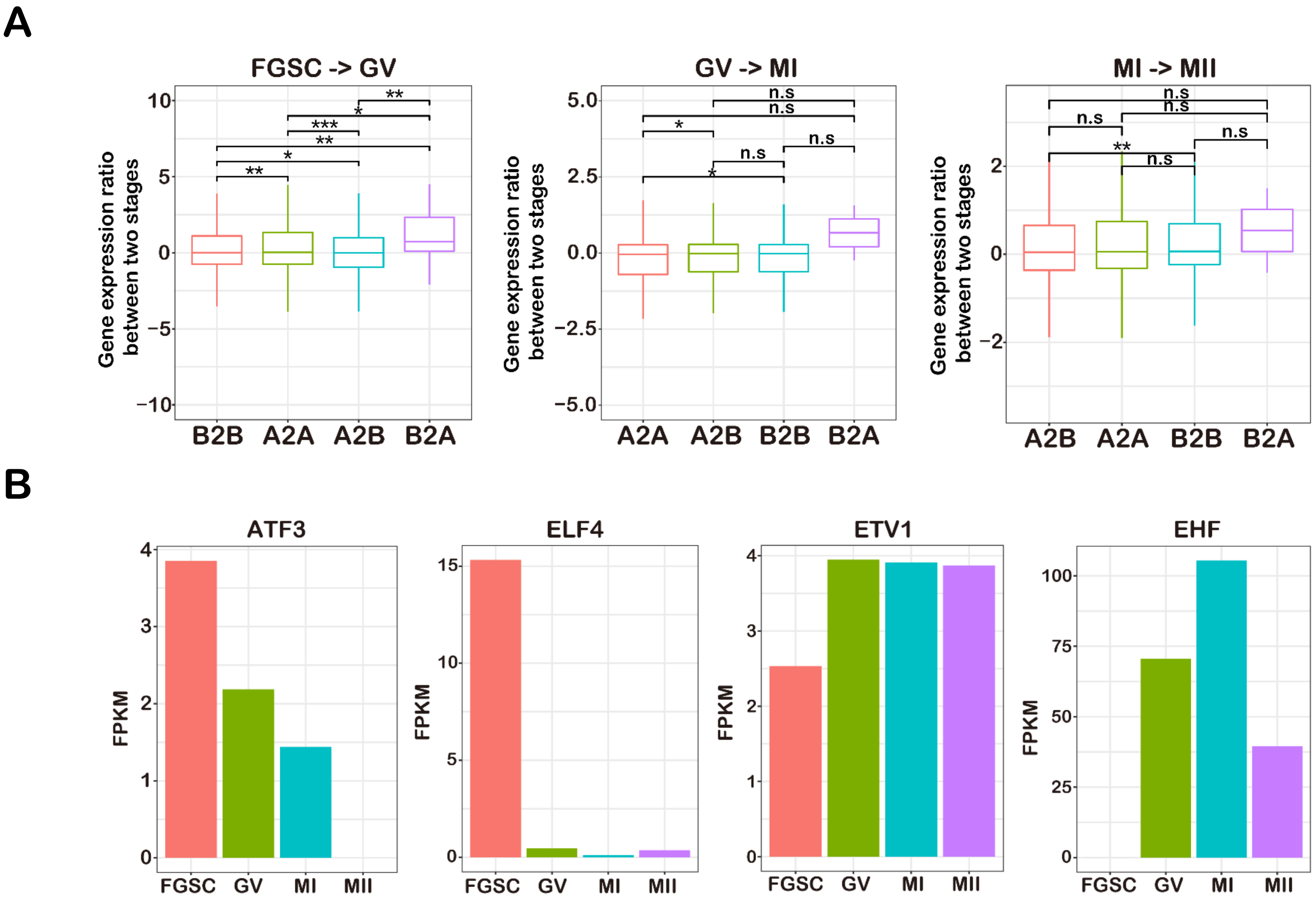

Figure S7. **Dynamic gene expression during FGSC development**

(A) Gene expression in the switch of TADs during FGSC development. (p value by Wilcoxon's test,*: p<0.05; **: p<0.01; ***: p<0.001)

(B) ATF3, ELF4, ETV1 and EHF expression during FGSC development.

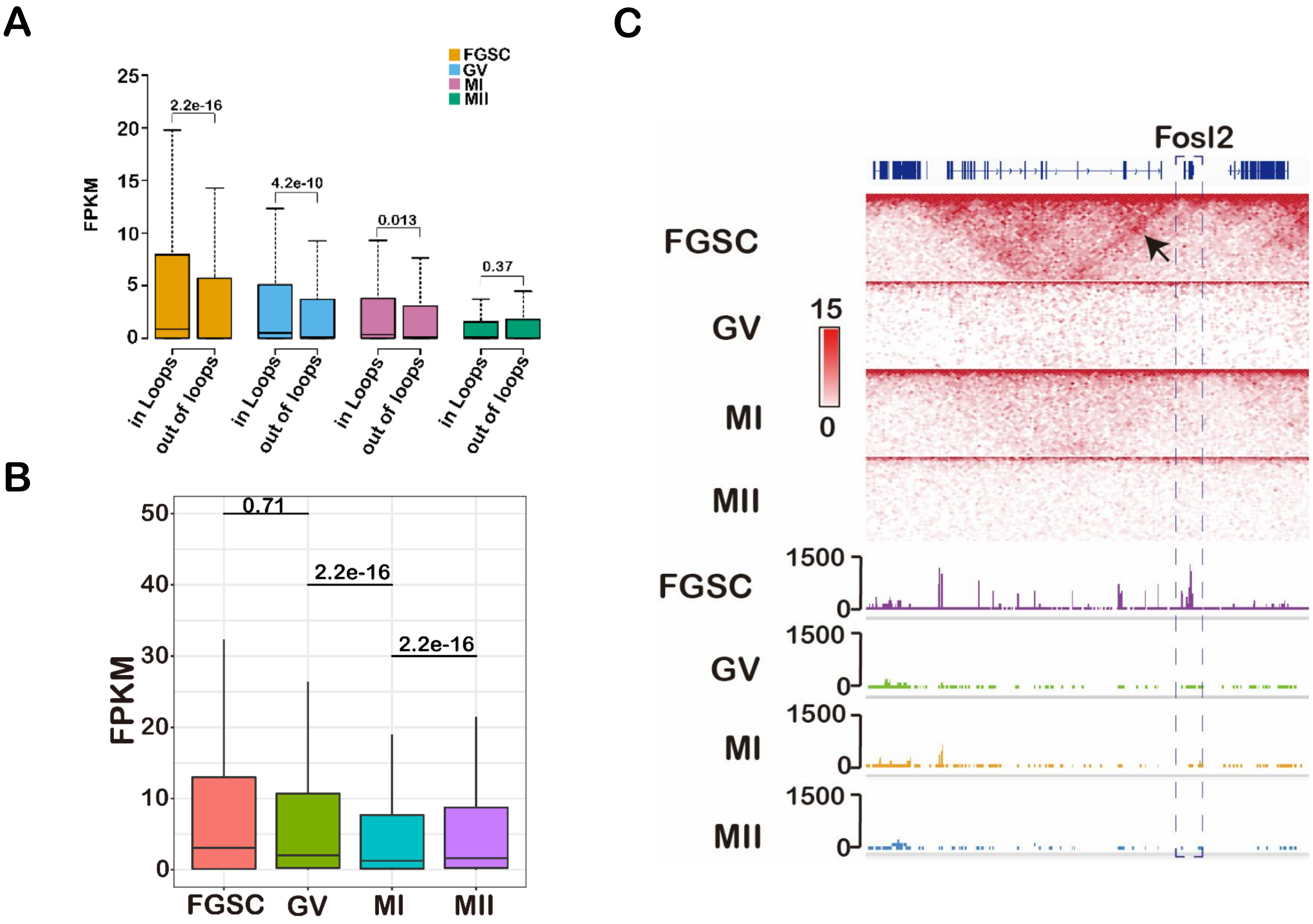

Figure S8. Chromatin loops were related with gene expression during FGSC development

(A) Gene expression within chromatin loops or without chromatin loops during FGSC development. (p value by Wilcoxon's test)

(B) Genes were inactivated during FGSC development. (p value by Wilcoxon's test)

(C) The dynamic changes of chromatin loops located in the promoter of Fosl2 during FGSC development.

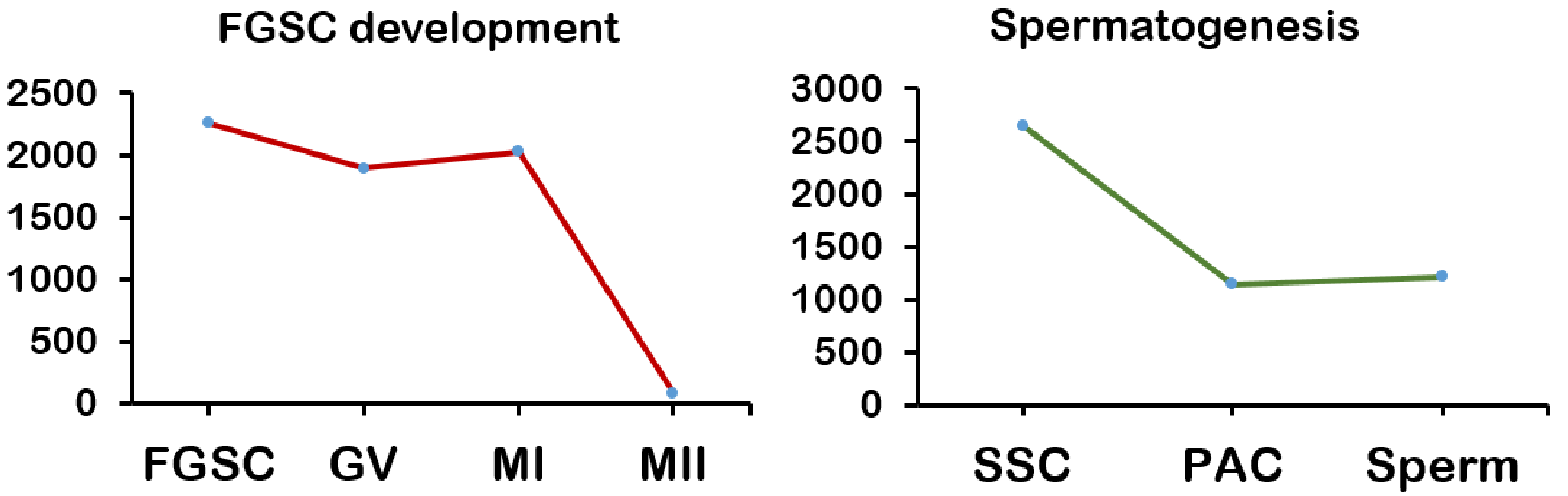

Figure S9. **TAD numbers during FGSC development and spermatogenesis**

The number of TADs was decreased during FGSC development, while it was recovered during spermatogenesis.
